# Contribution of Vegetation to the Microbial Composition of Nearby Outdoor Air

**DOI:** 10.1128/AEM.00610-16

**Published:** 2016-06-13

**Authors:** Despoina S. Lymperopoulou, Rachel I. Adams, Steven E. Lindow

**Affiliations:** Department of Plant & Microbial Biology, University of California, Berkeley, California, USA; University of Tennessee and Oak Ridge National Laboratory

## Abstract

Given that epiphytic microbes are often found in large population sizes on plants, we tested the hypothesis that plants are quantitatively important local sources of airborne microorganisms. The abundance of microbial communities, determined by quantifying bacterial 16S RNA genes and the fungal internal transcribed spacer (ITS) region, in air collected directly above vegetation was 2- to 10-fold higher than that in air collected simultaneously in an adjacent nonvegetated area 50 m upwind. Nonmetric multidimensional scaling revealed that the composition of airborne bacteria in upwind air samples grouped separately from that of downwind air samples, while communities on plants and downwind air could not be distinguished. In contrast, fungal taxa in air samples were more similar to each other than to the fungal epiphytes. A source-tracking algorithm revealed that up to 50% of airborne bacteria in downwind air samples were presumably of local plant origin. The difference in the proportional abundances of a given operational taxonomic unit (OTU) between downwind and upwind air when regressed against the proportional representation of this OTU on the plant yielded a positive slope for both bacteria and fungi, indicating that those taxa that were most abundant on plants proportionally contributed more to downwind air. Epiphytic fungi were less of a determinant of the microbiological distinctiveness of downwind air and upwind air than epiphytic bacteria. Emigration of epiphytic bacteria and, to a lesser extent, fungi, from plants can thus influence the microbial composition of nearby air, a finding that has important implications for surrounding ecosystems, including the built environment into which outdoor air can penetrate.

**IMPORTANCE** This paper addresses the poorly understood role of bacterial and fungal epiphytes, the inhabitants of the aboveground plant parts, in the composition of airborne microbes in outdoor air. It is widely held that epiphytes contribute to atmospheric microbial assemblages, but much of what we know is limited to qualitative assessments. Elucidating the sources of microbes in outdoor air can inform basic biological processes seen in airborne communities (e.g., dispersal and biogeographical patterns). Furthermore, given the considerable contribution of outdoor air to microbial communities found within indoor environments, the understanding of plants as sources of airborne microbes in outdoor air might contribute to our understanding of indoor air quality. With an experimental design developed to minimize the likelihood of other-than-local plant sources contributing to the composition of airborne microbes, we provide direct evidence that plants are quantitatively important local sources of airborne microorganisms, with implications for the surrounding ecosystems.

## INTRODUCTION

Many bacterial and fungal taxa can colonize aboveground plant parts (1). Because leaves are the most abundant of these plant parts, they harbor the largest numbers of such epiphytes (2). Despite the fact that the leaf surface is a hostile habitat due to variable water availability, high levels of incident UV irradiation, rapidly varying temperatures, and low nutrient availability, bacteria and yeasts are abundant colonists of the phyllosphere (3). It is estimated that the global phyllosphere bacterial community consists of as many as 10^26^ cells (4), given that the population size of bacterial epiphytes averages from 10^6^ to 10^7^ cells per cm^2^ of leaf (1).

Aerial plant surfaces are an open environment in which newly developing tissues typically harbor few epiphytes but on which a more robust community develops from immigrants transferred by a variety of mechanisms, including rain and deposition of airborne particles (2, 4, 5). Because of the high numbers of epiphytes on many plant parts, emigration from plants can be a substantial contributor of bacteria to nearby habitats. While rainfall commonly results in a net removal of bacteria from leaves, often resulting in their deposition onto soil (6, 7), wind-driven upward flux of bacteria from dry plants can be substantial (see, e.g., references 6 and 8). Accordingly, epiphytic microbes are readily recognized as a potential contributor to atmospheric microbial assemblages, but much of what we know is limited to qualitative assessments. For example, aerial emigration of bacteria from dry plants has been shown to be greater than that of either wet plants or soil (6, 8), and the concentration of epiphytes in the airborne microflora, based on the spatial gradient in numbers of airborne culturable cells or spores used to estimate the fluxes of bacteria and fungi away from plants, will be most pronounced nearby the plant (6–13). Lighthart (11) showed that the concentration of airborne microbial particles was as much as 10-fold higher near plants than in the bulk air away from the plants, especially under conditions in which release from the plant was stimulated by harvesting operations (11, 14), and Lindow and Andersen found that the rate of deposition of culturable bacteria onto surfaces was 5-fold higher near plants than away from such sources of epiphytes (15). Obviously, wind is an important factor mediating the dilution of airborne bacteria, and some have noted that under some conditions, microbes are diluted 10,000-fold within 30 m from their source, while the faster the microbes travel, the more the downwind sample resembles that of the upwind source (7).

Most of these studies of immigration and emigration of microbes from plants have utilized culturing to enumerate their abundance and identity. Current culture-independent techniques, however, are better able to assess the full diversity of organisms immigrating to or emigrating from plants and should thus provide a better estimate of these fluxes. Furthermore, much of the interest in phyllosphere microbiology research has focused on plant pathogens and specifically on the mechanisms that regulate their assembly, survival, and pathogenicity (16), although pathogens are only one component of a rich phyllosphere community. Recently, some studies have addressed the successional dynamics (17), the evolutionary associations between plants and phyllosphere bacteria (18), and seasonal (19) and geographical (20–22) features describing bacterial abundance and community composition on plants, and they have addressed the role of the plant in selecting for particular epiphytic communities (reference 4 and references therein).

To date, studies considering the contribution of epiphytes to the larger composition of airborne microbes are limited. Examinations of the microbial composition of air, performed using culture-independent methods, have usually been done at sites that are remote from abundant local vegetation or at relatively high elevations with low densities of vegetation (23–26). Nevertheless, these studies have shown evidence that taxa typically found in the phyllosphere are present in the air. Culture-independent studies of microbial composition in air samples likely detect the presence of cells, many of which may not be viable, originating even great distances away from the site of air sampling. While studies of airborne microbial communities demonstrate spatial and temporal variations (see, e.g., references 23, 24, and 26), most of these studies have not been designed to determine the sources of the atmospheric microbes. Some attempt has been made to identify sources of plant-derived airborne microbes by various meta-analysis approaches (24, 26), usually over relatively large spatial scales. Thus, while there is evidence that plants can be a source of some of the microbes found in outdoor air, few studies have addressed source-sink relationships between plants and airborne microbes and the relative magnitude and spatial scale of such exchanges.

Elucidating the source of microbes of outdoor air can inform basic biological processes, such as dispersal and biogeographical patterns seen in airborne communities (see, e.g., reference 27). Furthermore, recent studies have demonstrated that in many locations, outdoor air is a major contributor to the communities of bacteria and fungi found within indoor environments, such as residences (28–31). An understanding of plants as sources of airborne microbes in outdoor air might contribute to our understanding of indoor air quality; for example, many of the fungal spores responsible for allergies originate on outdoor plants. To test the model that plants serve as a major source of local airborne microbes and hence contribute to the microbial composition of air close to the plants, we compared the epiphytic microbial communities on vegetation with that of the nearby outdoor air. To simplify the interpretation of these relationships, we compared microbial communities in air collected simultaneously directly above vegetation of a single dominant plant species with that collected above an adjacent upwind nonvegetated area to estimate the magnitude of immigration of microbes from the source (vegetation) to the sink (air). We show, using culture-independent methods of community composition, that bacterial and fungal taxa common on the test plants were measurably increased in both absolute and relative abundance in air after passing over as little as 50 m of the source vegetation.

## MATERIALS AND METHODS

### Sampling.

We simultaneously sampled two air parcels, one within a vegetated area, and another at an adjacent upwind nonvegetated area approximately 50 m away. All sampling was done in the vicinity of Berkeley, CA, a coastal city on the eastern shore of San Francisco Bay subjected to persistent westerly winds off the Pacific Ocean. Many sampling sites were near the shoreline of San Francisco Bay in areas naturally colonized by Avena barbata and other introduced grass species (L01 to L04, L10, and L11) (Table 1; see also Fig. S1 in the supplemental material). Other sample sites were located in an urban area near the University of California campus, in which there were large plantings (>1 ha) of either tall fescue (Festuca spp.) (L05 and L09) or bell bean (Vicia faba) (L06 to L08). Very little vegetation was present in the urban area upwind (west) of these urban sampling sites. Upwind air samples were collected from either the shore of San Francisco Bay or a cement surface demarcating the western boundary of the vegetated sites within the urban area, while the downwind sites were simultaneously collected 50 m to the east. All air samples were collected on a 0.2-μm-pore-size, 47-mm-diameter sterile nitrocellulose filter that was preloaded into a sterile Nalgene analytical test filter funnel (Thermo Fisher Scientific) with a vacuum pump operated at 14 liters min^−1^ (∼51 kPa), approximately 1 m above the ground. The sampler was located 1 m above the ground and was operated for 65 min to collect a total of ∼1 m^3^ of air. Leaves of the predominant vegetation at each site were also collected at the time of sampling. Three replicate leaf samples (ca. 50 g) at each site were weighed and submersed in 100 ml of diethylpyrocarbonate (DEPC)-treated water in a Ziploc bag. Epiphytic microbes were removed from vegetation by sonication in an ultrasonic bath (Branson 5510) for 15 min. The water was then prefiltered through 10-μm-pore-size (47-mm diameter) polycarbonate membrane filters (EMD Millipore Corp.), and the cells within the filtrate were collected on a 0.2-μm-pore-size, 47-mm-diameter sterile nitrocellulose filter in the apparatus described above. The 10-μm-pore-size prefilter was chosen to exclude plant debris but include most fungal spores or cells.

**TABLE 1:** Samples that were processed from each location

Sample	Location	Location abbreviation	Upwind air sampling location	Downwind air sampling location	Plant
1	San Leandro Marina Park	L01	37°41′38.46″N, 122°11′12.39″W	37°41′36.59″N, 122°11′10.27″W	Festuca sp.
2	Miller/Knox Park shoreline	L02	37°54′34.28″N, 122°23′19.50″W	37°54′36.62″N, 122°23′18.80″W	Avena barbata
3	Berkeley Marina César Chávez Park	L03	37°52′15.14″N, 122°19′17.42″W	37°52′16.47″N, 122°19′17.30″W	Festuca sp.
4	Point Pinole State Park	L04	37°59′39.69″N, 122°21′36.85″W	37°59′39.64″N, 122°21′34.88″W	A. barbata
5	UCB campus	L05	37°52′17.77″N, 122°15′56.97″W	37°52′18.56″N, 122°15′56.33″W	Festuca sp.
6	UCB research field	L06	37°52′33.14″N, 122°16′3.26″W	37°52′33.78″N, 122°16′1.93″W	Vicia *faba*
7	UCB research field	L07	37°52′33.19″N, 122°16′3.45″W	37°52′33.78″N, 122°16′1.93″W	V. faba
8	UCB research field	L08	37°52′33.14″N, 122°16′3.26″W	37°52′33.78″N, 122°16′1.93″W	V. faba
9	UCB campus	L09	37°52′17.77″N, 122°15′56.97″W	37°52′18.56″N, 122°15′56.33″W	Festuca sp.
10	Berkeley shoreline	L10	37°52′13.07″N, 122°19′15.70″W	37°52′13.48″N, 122°19′13.84″W	Hordeum jubatum
11	Berkeley shoreline	L11	37°52′18.59″N, 122°19′20.62″W	37°52′19.03″N, 122°19′18.62″W	A. barbata

### DNA extraction.

DNA was extracted using a protocol previously described for air bioaerosols (28), which combines phenol-chloroform and the PowerSoil DNA isolation kit (Mo Bio, Carlsbad, CA, USA). The following two modifications were applied: (i) Miller phosphate buffer was replaced by the phosphate buffer of the FastDNA Spin kit for soil (MP Biomedicals), and (ii) all reagents were treated with propidium monoazide (PMA) (Biotium, Inc., Hayward, CA) at a final concentration of 10 μM in order to reduce DNA contamination, which can be an issue when deep sequencing is applied to low-biomass samples (32). One half of a filter was processed for air samples, while a whole filter was processed for plant samples; two extractions, each using one half of a filter, were pooled before the final cleaning/elution step for plant samples. DNA was eluted in buffer C6 in two steps, for a final volume of 70 μl.

### Library preparation and sequencing.

For bacteria, the V4-V5 variable region of the 16S rRNA gene was amplified with primers 515F and 806R (33), with barcodes on the reverse primer. For fungi, the internal transcribed spacer 1 (ITS1) variable region of the rRNA gene was amplified with the ITS1F and ITS2 primers, again with barcodes on the reverse primer (34). Air samples were amplified in a 2-step PCR; the 1st step consisted of 25 cycles and the 2nd of 8 to 12 cycles. Unlabeled reverse primers were used for the 1st step, and barcoded primers were used in the 2nd step. The PCR amplification reaction mixtures contained 0.6 units of Phusion high-fidelity polymerase (catalog no. F-530L; Thermo Fisher Scientific), 5× high-fidelity (HF) buffer, 200 μM dinucleoside triphosphates (dNTPs), a 0.2 μM concentration of each primer, 0.25 μg of bovine serum albumin (BSA), 5 μl of sample DNA (1st step) or 1 μl of PCR product (2nd step), and water for a 25-μl final reaction volume. Amplification was performed under the following conditions: 98°C for 30 s, followed by 25 or 8 to 12 cycles of 98°C for 10 s, 50 and 51°C for 30 s for bacteria and fungi, respectively, and 72°C for 30 s, and a final elongation step at 72°C for 10 min.

Plant DNA was amplified using the HotStarTaq Plus master mix kit (Qiagen, USA) under the following conditions: 95°C for 5 min, followed by 28 cycles of 94°C for 30 s, 50 and 51°C for 30 s for bacteria and fungi, respectively, and 72°C for 1 min, after which a final elongation step at 72°C for 10 min was performed. Peptide nucleic acids (PNAs) were added at a final concentration of 50 μM to block the amplification of mitochondrial PNA (mPNA) and plastid PNA (pPNA) DNA from the host (35). The PCR amplification reaction mixtures also contained 0.65 units of HotStarTaq, 10× buffer, 200 μM dNTPs, a 0.2 μM concentration of each primer, 0.25 μg of bovine serum albumin (BSA), 5 μl of sample DNA, and water for a 25-μl final reaction volume. A PNA annealing step at 78°C for 10 s was included before the primer annealing. Amplification of plant samples without PNA met with limited success, and thus, those samples are not included here. For those plant samples that did amplify successfully without PNA, the microbial community composition was similar to that of the paired samples that were treated with PNA (see Fig. S2 in the supplemental material).

After amplification, PCR products were examined in a 1.5% agarose gel, cleaned using Agencourt AMPure magnetic beads (Beckman Coulter, Brea, CA, USA), quantified with the Invitrogen Qubit HS double-stranded DNA (dsDNA) kit (Invitrogen, Carlsbad, CA, USA), and pooled in equimolar concentrations (10 nM). Bacteria and fungi were sequenced in two lanes of a MiSeq sequencer (paired 250-bp read lengths) at the Vincent J. Coates Genomics Sequencing Laboratory at UC Berkeley.

### Sequence processing.

Raw Illumina bacterial sequence reads were processed using mothur v.1.36 (36). Forward and reverse reads were paired based on their quality score. In subsequent screening, no ambiguous base calls were allowed, and reads with homopolymers exceeding 8 bp and shorter than 270 bp were removed. The data set was dereplicated, and unique sequences were aligned against the SILVA reference database (release 119) containing 137,879 bacterial small subunit (SSU) rRNA sequences (37). The data set was further denoised by running the “pre.cluster” command (38), and chimeras were removed with the UCHIME algorithm (http://drive5.com/usearch/manual/uchime_algo.html) (39), both of which were implemented in mothur. Unspecific amplification products (mitochondria, 1.1% of reads; chloroplasts, 7.9% of reads; Archaea and Eukarya, unknown domain) were removed, as well as singleton reads (see, e.g., reference 27). The remaining nonsingleton sequences were clustered into operational taxonomic units (OTUs) at a sequence divergence of 3% (40). High-quality sequences were classified (domain to genus level) using the curated SILVA database. Extraction and template-free PCR controls were processed as described below. To normalize the sequencing effort in all 30 samples without compromising the estimated amplicon diversity, all samples were randomly subsampled to the number of reads in the sample with the fewest reads (*n* = 4,714).

As OTU clustering in mothur requires sequence alignment, the fungal sequences were processed using USEARCH (41). The adapter sequences were removed using cutadapt (42) and then quality trimmed using Trimmomatic (43). Using USEARCH, forward and reverse reads were paired and then quality filtered (maximum expected error, 0.5). Unique sequences represented by two or more reads were clustered at 97% similarity, and then the resulting OTUs were checked for chimeras against the UNITE database, version dated 02.03.2015 (44). The same database was employed to assign taxonomic identification to representative sequences of the OTUs by using the BLAST algorithm, and taxa that did not match sequences in the database (*n* = 50) were excluded from the table. Fungal sequences in the samples were randomly subsampled to 1,200 reads, which eliminated downwind air VS3 (11 reads), upwind air VC10 (862 reads), and plant VP6 (4 reads). Too little DNA was recovered to obtain bacterial amplicons in upwind sample L01, while insufficient DNA was obtained from leaf washings of sample L07 to amplify fungal marker sequences.

Extraction controls (VEC2, VEC3, and VEC6) and no-template (PCR-negative) controls (NTCs) were processed with the samples. For fungi, no control samples were retained after rarefaction. For bacteria, the occurrence of OTUs retrieved from the NTCs was observed in a subsampled OTU table (*n* = 4,714). Those that were present in similar read numbers in the environmental samples (plants, *n* = 20; air, *n* = 32) and in the extraction controls were removed from the full OTU table. For those OTUs in NTCs (OTUX_NTC_) that were present in the environmental samples at numbers less than five times the number of sequences in the sample (plants, *n* = 9; air, *n* = 3), the number of reads was subtracted from the number of reads in the environmental samples and in the extraction controls from the full OTU table, by accounting for library size differences, for an OTUX in a sample (S) as follows: the final number of sequences of OTUX_S_ = the number of sequences of OTUX_S_ − (sample S library size/NTC library size) × the number of sequences of OTUX_NTC_.

Further correction by relative subtraction, as described above, accounted for the presence of common OTUs between extraction controls and environmental samples (VEC2, *n* = 25; VEC3, *n* = 31; and VEC6, *n* = 11) that had not been removed as part of the correction for the NTCs, and therefore their presence is related to the extraction and not to the amplification. We assumed that the correction should take place for the NTCs first, since both the environmental samples and the extraction controls were amplified under the same conditions. The “corrected” OTU table was subsampled using 3,801 sequences.

### Quantitative PCR.

Quantitative PCR on an ABI 7300 was employed to determine the microbial biomass of each sample. Bacterial biomass was determined based on the quantification of the 16S rRNA gene copies using the primer pair 341F and 534R (45). Data were retrieved at 60°C, and all reactions concluded with a melting curve starting at 60°C, with an increase of 0.5°C up to 95°C to verify amplicon specificity (a specific temperature between 85.50 and 86.50°C). Fungal biomass estimates relied on the FF2 and FR1 primers of the 18S rRNA gene (46). Each sample was measured in duplicate, and negative controls (NTCs) were included. Standard curves for each assay were obtained using serial 10-fold dilutions of plasmids containing a fragment of either the Escherichia coli ATCC 2592216S rRNA gene or an insert of the 18S gene of Penicillium purpurogenum. Amplification efficiency (*E*) was calculated using the slope of the log standard curve given by the ABI 7300 software, as follows: *E* = −1 + 10^(−1/slope)^. Efficiencies were 91% (*R*^2^ = 0.979) for bacteria and 96% (*R*^2^ = 0.990) for fungi. The results are reported as 16S or 18S gene copy numbers.

### Data analysis.

Alpha diversity estimators (e.g., Shannon-Wiener [H], the inverse Simpson [1/*D*], the richness estimator S_chao1_, and the Berger-Parker index) were calculated in mothur and R version 3.2.2 (R Development Core Team; http://www.R-project.org [47]), by relying on the base and the Phyloseq packages (48). The ggplot2 R package (49) was used for plotting. Pairwise dissimilarities between communities were calculated in mothur for bacteria and in the vegan package in R (50) for fungi using the Bray-Curtis index and were visualized via nonmetric multidimensional scaling (nMDS) in R. In addition, samples were visualized using hierarchical clustering based on the same dissimilarity index and were plotted with the package ampvis in R (51). To estimate the robustness of the ordination mentioned above, we used analysis of similarities (ANOSIM), a method that is similar to the Mantel test but appropriate for categorical environmental variables. We examined the differences in microbial assemblages among sites (location) and among habitat types (upwind air, downwind air, and plants). To assess the explanatory power of habitat type and site (location) on community composition and membership, we used a permutational multivariate analysis of variance (PERMANOVA) implemented by ADONIS in the vegan package (50), based on 1,000 permutations. The weighted Bray-Curtis index, which is highly resistant to singletons, the qualitative Jaccard index, and the weighted Canberra index, which emphasizes rare taxa, were used as indices to determine the community distance matrix (see Table S1 in the supplemental material).

A least-squares linear regression analysis of the difference between the proportional abundances of each OTU in downwind air (DW) and upwind air (UW) origins (*d*_Air_ = DW − UW) against the log-normalized proportional abundance of this OTU in the plants was performed. Only OTUs that were recovered from plants and with a proportional abundance of ≥0.1% were considered (bacteria, *n* = 716; fungi, *n* = 537). Because this correlation includes only those OTUs on plants, it shows the potential of plant-associated OTUs to act as sources of enrichment in abundance for the downwind air. Sites for which sequencing results were not available for all three habitats were not included in the analyses (bacteria, L01 and L04; fungi, L06, L07, and L10).

All statistical analyses were conducted at a *P* value of 0.05. Source tracking (52) was executed using SourceTracker version 0.9.5, in which air samples were classified as “sink” and plants as “source.”

### Nucleotide data accession number.

The raw sequence data of bacterial and fungal amplicons were deposited into NCBI's Sequence Read Archive (SRA) under study accession no. SRP065913.

## RESULTS

### Analysis and taxonomic overview.

Samples of dominant plants and two simultaneous air samples, one above (∼1 m) a vegetated area (downwind air) and another at an adjacent upwind nonvegetated area (upwind air) approximately 50 m away, were collected at 11 locations near the eastern shore of San Francisco Bay, and the microbial communities recovered were assessed by direct amplicon sequencing. The specific locations and dominant plant vegetation in each site are described in Table 1. Approximately 2.3 million paired Illumina reads of bacterial amplicons for the 46 samples (5 control samples, 1 mock community sample, and 4 miscellaneous samples not included in the analysis) were obtained. After quality processing, 903,535 reads comprising 18,116 unique sequences were clustered into 4,383 OTUs. After either removal of contaminant OTUs or correction for the abundances of OTUs also found in controls by relative subtraction and after removal of control samples, 703,896 reads remained. Approximately 2.1 million paired fungal amplicon reads were obtained. After quality processing, 508,964 reads comprising 79,704 unique sequences were clustered into 4,704 nonchimeric OTUs.

The diversity indices for bacteria and fungi are summarized in Table S2 and Fig. S3 in the supplemental material. For bacteria, Shannon and inverse Simpson (InvSimpson) diversity indices were higher for the upwind air than for the plant samples (Mann-Whitney pairwise test with Bonferroni correction, *P* < 0.05), but there were no differences in diversity between the downwind air and plant epiphytes.

**FIG 1:**
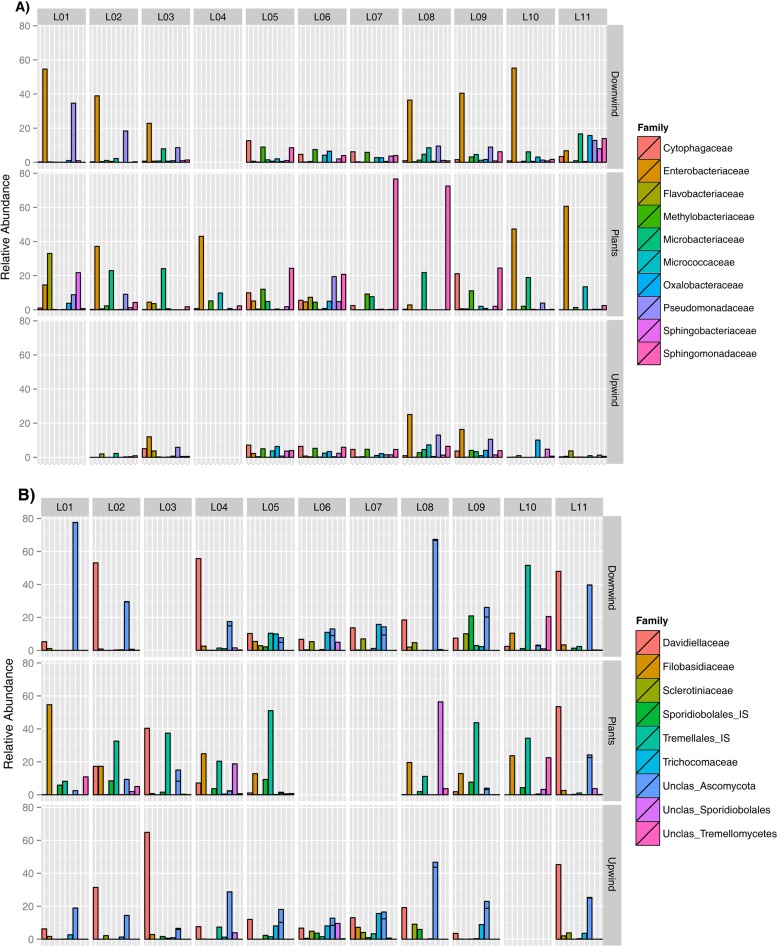
Relative abundances of the most pronounced families by location (horizontal axis) and by habitat (right vertical axis) for bacteria (A) and fungi (B). Unidentified families of fungi have been collapsed to the next known taxonomic level (usually the order level). IS, insertion sequence; Unclas, unclassified.

Proteobacteria was the dominant bacterial phylum on plants, comprising 66.5% of the reads, with the *Alpha*- and *Gammaproteobacteria* classes accounting for approximately 33% each, followed by Actinobacteria (16.4%) and Bacteroidetes (11.7%). Proteobacteria also were more abundant in downwind air (61.6%) than in upwind air (49.3%), with the Gammaproteobacteria class enriched (39.7% of sequences in downwind air samples against 22.4% in upwind air samples). It is noteworthy that the three phyla accounted for >92% of the sequences in all three sample origins, with Firmicutes (Bacilli) also accounting for 16.9% of the sequences in upwind air parcels. Several families, including Sphingomonadaceae (21.1%), Enterobacteriaceae (20.2%), Microbacteriaceae (9.2%), Methylobacteriaceae (4.5%), Flavobacteriaceae (4.1%), Pseudomonadaceae (3.9%), and Cytophagaceae (3.8%), were dominant on plants (Fig. 1A). The families that are generally associated with plants, such as Enterobacteriaceae, Methylobacteriaceae, Pseudomonadaceae, and Sphingomonadaceae, were proportionally more abundant in downwind air than they were in upwind air. For example, Enterobacteriaceae accounted for 25.7% of the reads in downwind air, while they comprised only 6.4% of the reads in upwind air. Similarly, Pseudomonadaceae and Microbacteriaceae, comprising 9.5% and 3.6% of the reads in downwind air, respectively, were about 3- and 4-fold higher, respectively, than the number of reads in upwind air parcels. No enrichment of epiphytic taxa was identified in downwind air at sites L08 and L09 (Fig. 1A), where the wind direction was irregular, occasionally blowing from the east rather than from the west, as at all other sites. Despite their dominance on plants, Sphingomonadaceae in downwind air were less enriched than several other epiphytic bacterial taxa.

**FIG 2:**
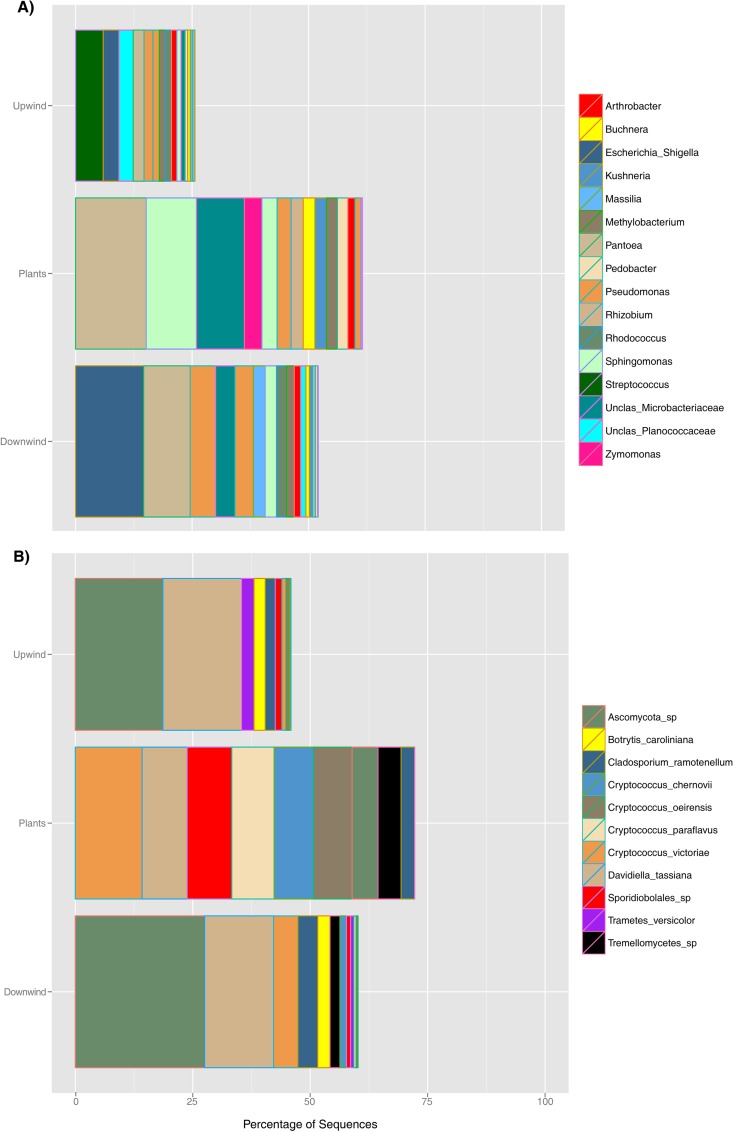
Relative abundances of the top 18 bacterial OTUs (spread among 16 taxonomic affiliations, with Pseudomonas and Sphingomonas having 2 affiliated OTUs each) (A) and the top 11 fungal OTUs (B) recovered in each habitat. The OTUs included are those present at an abundance of ≥0.1% of the reads for a given sample and together account for ≥50% of all sequences recovered.

Several bacterial taxa were highly dominant on leaves, with 18 OTUs accounting for 47.7% of the sequences recovered from all three sample origins. Similar to observations at the family level, the most abundant epiphytic OTUs were also the most enriched in downwind air relative to upwind air. These OTUs were affiliated with genera, such as Pantoea, Sphingomonas, and Pseudomonas, that are commonly found on plants (Fig. 2A). For example, Pantoea accounted for 24% of the sequences recovered from plants and was almost 2-fold more abundant in downwind air (19.1%) than in upwind air (9%). Likewise, Sphingomonas (OTU7), representing 19% of the sequences recovered from plants, was more abundant in downwind air (4.7%) than in upwind air (3.6%), while Pseudomonas also was about 1.5-fold more abundant in downwind air. An unclassified Microbacteriaceae OTU that was relatively abundant on plants (14.8% of recovered sequences) was found in a proportion that was 2.4-fold greater in downwind air than in upwind air.

Characteristic airborne fungi, including Cladosporium, Trametes versicolor, and Penicillium, were found in air samples, while fungal epiphytes were dominated by yeasts. Specifically, 96.9% of the sequences were distributed between Ascomycota (54.3%) and Basidiomycota (42.5%). Davidiellaceae were particularly abundant in all three sample origins (21% upwind, 22% downwind, and 13.4% on plants). However, some fungal families were substantially enriched in downwind air relative to upwind air, including Filobasidiaceae (1.9-fold) and Tremellaceae (the sexual and asexual forms of Cryptococcus, respectively), and unidentified families within the Sporidiobolales (1.7-fold), Tremellales (4.7-fold), and Tremellomycetes (9.7-fold) (Fig. 1B). Eleven of the most abundant fungal OTUs in all samples accounted for 58.6% of the sequences recovered. The most abundant taxa on plants included different species of Cryptococcus, such as C. victoria (14.1%), C. chernovii (8.4%), C. paraflavus (8.9%), and C. oeirensis (8.1%). Among these species, C. chernovii and C. paraflavus were more abundant in downwind air than in upwind air. Likewise, two sequences associated with Cladosporium and one with Botrytis were also more abundant in downwind air than in upwind air, increasing from 2%, 17.2%, and 2.2% to 4.2%, 27.4%, and 2.6%, respectively (Fig. 2B). While the abundance of several different fungal taxa was substantially higher in downwind air than in upwind air, with the exception of a Cladosporium sp., their absolute read abundance even in downwind air was relatively low. Furthermore, unlike with the taxa of epiphytic bacteria, many of the most abundant fungal taxa on plants were not found to be enriched in downwind air. Thus, the contribution of fungal epiphytes to the air seems to be more taxon specific than the contribution of bacterial epiphytes.

### Quantity of microbes.

Large temporal and spatial variations in the gene copy numbers of both bacteria and fungi per cubic meter of air were found by quantitative PCR (Table 2). The concentration of fungal biomass in downwind air was greater than that in upwind air, with only one exception. However, the magnitudes of the increased concentration in downwind air relative to that in upwind air were highly varied; in one sample, fungal biomass was 1.5-fold greater, while in another sample, it was as much as 440-fold higher. The concentration of bacteria in downwind air was generally higher than that in upwind air and fell within a narrow range of effect (from 0.4- to 8.6-fold).

**TABLE 2:** Abundances of bacteria and fungi determined by quantitative PCR in upwind and downwind air samples collected at a given site^*a*^

Site	Abundance of bacteria in:		Abundance of fungi in:	
	Upwind air	Downwind air	Upwind air	Downwind air
L01		1,070,000		13,000
L02	37,000	311,000	8,000	47,000
L03	260,000	1,580,000	9,600	2,170,000
L04				231,000
L05	687,000	1,260,000	46,000	88,000
L06	262,000	31,600	88,000	109,000
L07	1,180,000	291,000	70,000	102,000
L08	246,000	516,000	10,000	31,000
L09	29,700	58,300	9,000	6,000
L10	466,000	2,750,000		398,000
L11	158,000	1,460,000	32,000	14,000,000

a Abundances are represented as numbers of gene copies per cubic meter of air.

### Plants as sources of airborne microbes.

**FIG 3:**
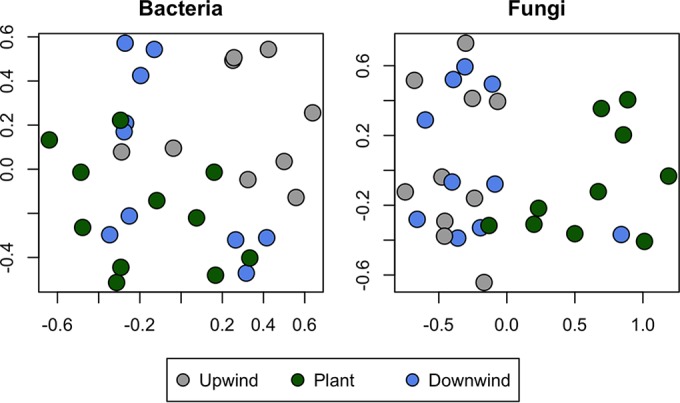
Compositional comparison of samples in a nonmetric multidimensional (nMDS) scaling plot in two dimensions, constructed from a Bray-Curtis distance matrix of OTU abundances and color-coded by sample origin.

Several analytic approaches revealed the extent to which plants served as quantitatively important sources of airborne microbes. Broad patterns of similarity in taxonomic composition were observed between epiphytic communities and microbes in downwind air in nonmetric multidimensional scaling (nMDS) plots exploiting the Bray-Curtis index (Fig. 3). For bacteria, upwind air samples grouped separately from those of plants and downwind air samples in most cases, while communities on plants and downwind air could not be readily distinguished (Fig. 3). In contrast, fungal taxa in air samples were more similar to each other than to fungal epiphytes on plants. Hierarchical cluster analysis (see Fig. S4 in the supplemental material) showed a pattern similar to that observed in the nMDS, with the plants grouping separately from the air samples where fungi were concerned, except plants and downwind air samples from sites L10 and L11, which clustered together. For bacteria, upwind air samples from L02, L03, L10, and L11 formed a cluster out of the main dendrogram, showing a clear demarcation from their respective downwind samples. Some plants also formed a distinct group, and generally, downwind air samples tended to resemble each other more than they resembled their respective plant sources, regardless of site.

The differences in microbial assemblages among sites (location) and among habitat types (upwind air, downwind air, and plants) were also compared. For both bacteria and fungi, differences in the community composition were explained better by habitat type (ADONIS, *P* < 0.01 for all indices tested; see Table S1 in the supplemental material) than by sampling location, such that habitat type accounted for 14% of the variation in bacteria and 16% of the variation in fungi, based on the Bray-Curtis index. We also performed ANOSIM with the above-mentioned three *a priori*-defined groups, upwind air (UW), plants (P), and downward air (DW), as a test of significance of differences in community assemblages in these various habitats. The value of the ANOSIM *R* statistic ranges from 0 (no separation) to 1 (complete separation of the groups tested). For bacteria, plants and downwind air were more similar to each other (ANOSIM, *R* = 0.20, *P* < 0.007) than plants and upwind air were (ANOSIM, *R* = 0.40, *P* < 0.001). These comparisons became more robust when the samples affected by the uneven wind direction were partially or fully removed; when either L08 or L09 was removed, the P-UW ANOSIM *R* was 0.47 (*P* < 0.001), while the P-DW ANOSIM *R* was 0.16 or 0.20, respectively (*P* < 0.05), corresponding to stronger separation or stronger connection, respectively, between the habitats compared. Removal of both sites resulted in an *R* value of 0.61 (*P* < 0.001) for the P-UW comparison and an *R* value of 0.17 (*P* < 0.047) for the P-DW comparison. Nevertheless, upwind and downwind air had a close relationship (*R* = 0.24, *P* < 0.054), as would be expected if epiphytic microbes were added to otherwise-similar air parcels. In agreement with the results of studies of bacterial taxa, fungi in downwind air were more similar to those on plants (*R* = 0.41, *P* < 0.05) than those in upwind air were to those on plants (*R* = 0.54, *P* < 0.001). However, there was no distinction between fungi found in upwind and downwind air (ANOSIM, *R* = −0.07, *P* < 0.93).

We implemented the SourceTracker algorithm to determine the portion of each sink community (air) that could be attributed to a putative plant source (Table 3). Plants generally were predicted to be a weak source of airborne fungi, ranging from less than 1% to 14%, although they were more likely to be a source of fungi in downwind air than in upwind air. The proportion of airborne bacteria that were presumably of plant origin ranged widely between samples (from 0.4% to 50.8%). Some of this variable contribution of plants to airborne microbes was attributable to the mass of the plant at a given site. For example, while young V. faba plants in samples L06 and L07 (less than 30 cm in height and presumably having relatively low epiphytic populations, as in other studies [5]) were predicted to contribute only 0.4 and 0.7% of the sequences observed in downwind air, respectively, more mature plants at this site sampled after 2 months that were as tall as 1.5 m (L08) and had larger epiphytic populations contributed as much as 30% of the sequences recovered from downwind air samples. As was expected, the contribution of plants to the bacterial composition of downwind air was significantly more than that to upwind air (*t* test, *P* < 0.05).

**TABLE 3:** Percentages of bacteria and fungi found to be contributed by plant sources in a given upwind and downwind air sample

Site	% contributed by plants			
	Bacteria		Fungi	
	Upwind air	Downwind air	Upwind air	Downwind air
L01		2.7	0.6	1.4
L02	0.06	5.3	1.1	2.0
L03	0.58	17.4	3.6	
L04			16.2	12.2
L05	4.1	8.6	1.3	10.6
L06	0.61	0.4	3.1	2.3
L07	0.95	0.7	9.3	1.4
L08	41.46	30.5	0.9	2.7
L09	34.05	42.4	0.4	0.2
L10	0.16	21.9	0.7	97.7
L11	1.33	50.8	0.7	14.0

**FIG 4:**
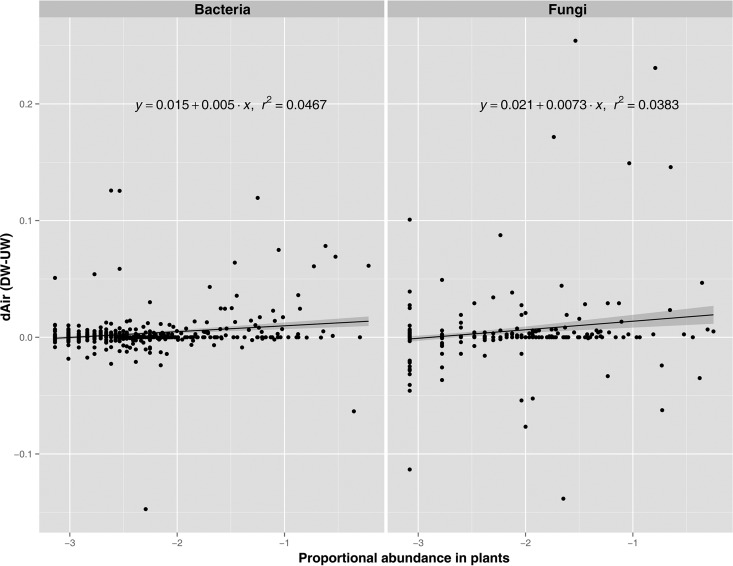
Least-squares linear regression analysis of the difference in the proportional abundances of a given OTU found in downwind air and in upwind air (*d*_Air_ = DW − UW) against the log-normalized proportional abundance of this OTU on plants at the sample site. The proportional abundances on plants (*x* axis) were log transformed [log(*x* + 1)] to better illustrate this relationship given the wide range of proportional abundances of the OTUs. Only OTUs that represented ≥0.1% of the reads in a given plant sample were included (bacteria, *n* = 716; fungi, *n* = 537). Two OTUs having unexplainably high abundances in one sample were omitted (bacteria, OTU1 in L10; fungi, OTU3 in L01). Sample sites for which sequences from all three habitats were not available were not included (bacteria, L01 and L04; fungi, L06, L07, and L10).

Given that many taxa were found both in upwind and downwind air, probably due to their release from plants and a variety of other sources at distant upwind sites, the quantitative contribution of plants to the downwind air was assessed by a least-squares linear regression analysis that took into consideration the relative abundance of a given taxon on the putative plant source (Fig. 4). The difference in the proportional abundances of a given OTU between downwind and upwind air was regressed against the proportional representation of this OTU on the plant. The slope for both bacteria and fungi was found to be positive (*P* < 0.001), revealing that if epiphytes were indeed a substantial source of airborne bacteria, as expected, those taxa that were most abundant on plants contributed proportionally more to the community in downwind air than to that in upwind air. That is, the more abundant a given bacterial or fungal taxon was on plants, the more likely that taxon was to be enriched in downwind air. However, variation was great, and the overall slope had little explanatory power (Fig. 4).

## DISCUSSION

This work shows that epiphytic microorganisms on plants contribute substantially to the composition of bacteria in the air nearby those plants, with a lower observed effect for fungi. While it is known that microorganisms can escape plants by both passive and active means (where they would then be entrained in the local air), no prior studies had addressed the quantitative importance of these processes to the overall microbial composition of nearby air.

### Experimental design and identifying local epiphytic sources.

It was expected that atmospheric samples made over continental regions, harboring a variety of plant species, would contain taxa that had originated on these plants. Unfortunately, the uncertain origin of continental air masses and the likelihood that they may entrain bacteria from even distant plants make analysis of source-sink relationships in such a setting difficult. Several studies have explored the microbial diversity of outdoor air as a function of elevation (25), land use types (24), season (26), and spatial (city) scale (23). While these studies have revealed considerable spatial and temporal variations in microbial communities, they were not designed to distinguish local from more distant sources of airborne microbes. While taxa such as Enterobacteriaceae, Sphingobacteriaceae, Sphingomonadaceae, Oxalobacteraceae, Burkholderiaceae, Moraxellaceae, Actinomycetales, and Flavobacteriaceae, which are typically seen on plants, have been noted in culture-independent analyses of the atmosphere and have been attributed to a plant origin (see, e.g., references 23 and 26), the spatial context in which they may have been entrained into the air could not be ascertained.

The experimental design utilized in this study was developed to optimize our ability to quantify the contribution of local epiphytic microbes to the composition of airborne microbes. In an attempt to isolate the contribution of those plants under study, experimental sites in which few or no plants were present in habitats upwind of the terrestrial sample site were selected. Contrasting terrestrial sources of airborne microbes were obtained by sampling air that had passed over large extents of either marine water or dense urban development in which few or no plants were present. Furthermore, the vegetated areas over which air had passed in this study were densely covered with a predominant plant species, minimizing soil as a contributor of microbes to the air that had passed over these areas. The assumption that microbial inhabitants of plants would be distinguishable from those originating in aquatic environments was made.

### Release of plant epiphytes as bioaerosols.

While the abundance of both airborne bacteria and fungi increased dramatically in air that had passed over as little as 50 m of vegetation, such that both bacteria and fungi were nearly always much more abundant (by 2-fold to more than 10-fold) in air that had passed over vegetation than in air immediately upwind of the vegetation (Table 2), the observed influence of bacterial epiphytes was much greater than that of fungi. This may be for a variety of reasons. The absolute abundance of fungi in the air was generally much lower than that of bacteria (Table 2), and epiphytic fungi usually establish much lower population sizes on healthy plants than bacteria (reference 53 and references therein). Thus, their lower apparent source strength for the atmosphere than that of bacteria is consistent with a lower overall presence as epiphytes. The particular composition of the fungal epiphytes for the plant species samples may also have played a role, since it is likely that there are taxon-specific differences in how readily epiphytes are passively transported by air away from the plant surface.

It was somewhat surprising that certain bacterial and fungal taxa were apparently more likely to have been released into the air from the leaf surface than others (Fig. 1 and 2). Examples of bacterial taxa that were preferentially released included Pantoea, Sphingomonas, and Pseudomonas, while Zymomonas, Kushneria, Pedobacter, and Rhizobium were underrepresented in the air relative to their numbers on leaves (Fig. 2). It is noteworthy that Sphingomonas has been commonly found as a bioaerosol in a wide variety of terrestrial environments, including urban (23, 26, 54), rural (26), forest (18), and high-alpine (55) ecosystems. Both its frequent abundance on leaves and its apparent high efficiency of release from plants might account for Sphingomonas's ubiquity in the air. Cryptococcus chernovii and two Cladosporium spp. were the fungal taxa that were apparently most efficiently released from plants, while other Davidiella and Cryptococcus spp. were not enriched in the air downwind of plants, despite their relatively high epiphytic populations. While yeasts are known to be common fungal epiphytes (56), studies on epiphyte release have focused on spore formers (9, 10). In outdoor air, yeasts are much less abundant than filamentous fungi (57). Two filamentous fungi that were found to be more abundant in downwind air were Cladosporium and Botrytis, both phylloplane fungi common in outdoor air. Their conidia, which are produced in massive numbers, easily become airborne through passive means (58). Although particle size is an important parameter determining movement through the air, the relationship between the size of the organism or its propagating body and its likelihood of release from plant surfaces and transit has not been established. For the fungal taxa identified here, there was great overlap in particle size; for example, the yeast Rhodotorula has cells that are 2 to 6 by 6 to 14 μm, while Cladosporium spores are approximately 3 to 7 by 2 to 4 μm (59).

The apparent bias in the release of different taxa from leaves may be driven by several factors, such as the sizes of aggregates that they form (4), differences in the sites in which they localize on leaves (2, 4), and possible differences in their adhesiveness to plant surfaces (see, e.g., reference 60). It is presumed that bacteria, yeasts, and many fungi are released passively from the plant surface (7). The analysis of atmospheric particles containing viable bacteria has revealed that they are generally much larger than individual cells (61, 62). As such, bacteria and other microbes are probably usually present as part of a cellular aggregate in the air. Epiphytic bacterial as well as yeast populations are also highly aggregated on leaves (4, 63, 64). For example, the majority of the cells of a given species, such as Pseudomonas syringae, are found in relatively large aggregates (>100 cells) (63). It is tempting to speculate that the interaction of microdrafts of air with the leaf surface may more readily dislodge such bacterial aggregates than more solitary cells. Also, microbes are generally not randomly distributed spatially on leaves and instead are preferentially located at the edges of leaves and near veins (2, 63), sites where turbulent air might be more likely to cause their removal. Microbial communities, including bacteria, are also spatially segregated on leaf surfaces (64). Spatial segregation may be driven by differences in local resource availability or composition (65). Thus, cells of various taxa may experience different microenvironments on leaves because of the different locations that they inhabit. More-detailed studies of the mechanisms of release of various bacterial and fungal taxa from plants should prove insightful.

### Connections between airborne epiphytes and other ecological systems.

The contribution of epiphytic species to the air nearby was supported by the important finding that the proportional enrichment of a given taxon in downwind air compared to that in upwind air was generally directly related to its relative abundance on the plant (Fig. 4). That is, those taxa that were most abundant on plants were also the most highly enriched in the air, as expected if plants are the source of the airborne microbes. The finding that families such as Sphingomonadaceae and Methylobacteriaceae were among the most dominant epiphytic bacteria and the taxa most enriched in downwind air in this study supports the conjecture made in other studies of airborne microbes (e.g., references 24 and 26) that plants are the origin of such taxa.

The results of this study support a model in which microbes from abundant sources on plants are added locally to microbes originating from more-distant sources in an air mass. It seems likely that informative molecular markers, such as the small-subunit rRNA gene used to describe bacterial assemblages and the ITS region used to describe fungal communities, are quite persistent in airborne microbes. Upon entrainment into the air, such microbes are expected to rapidly dehydrate, a process that promotes the longevity of DNA markers. Thus, a given parcel of air is expected to harbor a wide collection of microbes originating from various terrestrial and aquatic habitats over which that air has passed. Given the rapid transit time of air parcels over most parts of the earth, the origin of many microbes might be quite distant from where they are detected at a given site. Mixing of air parcels is expected to substantially reduce the concentration of microbes relative to that near their source (7). Various microbes from various points or area sources therefore contribute to a relatively uniform and dilute microbial load in a given parcel of air. However, upon encountering a strong source of airborne particles, such as plant surfaces, the local concentration of microbes is expected to be higher than, and distinct from, that of the collection of microbes that originated from more-distant sites. In many ways, this process is analogous to the dispersal of smoke away from point sources; while the atmosphere at a given site harbors a very dilute milieu of smoke particles from fires that occurred far away, the concentration of smoke is high only near a local fire, and its concentration decreases visibly with distance. Thus, while a given air parcel might be expected to harbor microbes of plant origin, aggregating microbes from various plants over which that parcel had passed, a signature of the microbes found on local vegetation should be readily visible given their higher concentration due to a lack of dilution of the air parcel close to the site of its release. As discussed earlier, the efficiency by which a given source of microbes can contribute such particles to the air is influenced by both the efficiency of the microbes' release from their origin and their concentration at the origin. While marine sources of airborne microbes, such as wind-wave and wind-shore interactions or bursting of bubbles, have been demonstrated (66), microbes apparently can escape plant surfaces much more readily, given that the numbers of microbes in coastal aerosols are relatively low compared to those in aerosols over terrestrial/vegetated areas (13).

The relatively high airborne concentrations of bacteria and fungi from local vegetation might create a feedback loop in which the vegetation at a given site shares a metacommunity that is distributed via airborne particles. Thus, plants may serve not only as sources of airborne particles but also as sinks. This depositional process might explain why a substantial location effect is seen in epiphytic bacterial communities. That is, the composition of bacterial communities on a plant species at a given site often differs from that of the same species at a distant site (21, 67) but is somewhat similar to that seen on other plant species at that site (67). The assembly of epiphytic communities on a given plant therefore might be driven not only by the features of a host plant but also by the identities of other plant species that might contribute an immigrant airborne inoculum.

There are numerous implications of the finding of a substantial plant contribution of microbes in outdoor air to those in the built environment. In many residences and other components of built environments that are relatively permeable, the composition of indoor air microbial communities is strongly influenced by that of the outdoor air at that location (30, 31, 68). Parcels of outdoor air carrying microbes can enter into the built environment in various ways. Furthermore, humans, animals, and other objects that enter the built environment can serve as vectors for microbes found outside the building (69). Irrespective of the method of entry of microbes, the contribution of microbes found outdoors, including in the air, should increase with their local abundance. Penetration of air parcels from outdoors through doors and windows, for example, can enable the introduction of both bacteria and fungi, particularly because their abundance in outdoor air is typically higher than that in indoor air (30, 70). In such a scenario, factors that determine the composition of microbes in outdoor air will contribute disproportionately to that in indoor air. Given that this study has shown that local vegetation strongly influences the composition of outdoor air locally, we extrapolate to propose that outdoor vegetation near buildings strongly influences the composition of microbes in indoor air. Thus, given that plant species identities determine in part their epiphytic microbial communities (4, 71, 72), the microbial composition of indoor air is driven at least partially by the abundance and identity of vegetation nearby the building. In addition, because of the apparent regional metacommunity of epiphytic microbes discussed above, the biogeographical signatures seen in indoor fungi (27, 28, 73, 74) may be driven at least in part by local or regional plant communities.

## Supplementary Material

Supplemental material
