# Supplementary material for: Contribution of Vegetation to the Microbial Composition of Nearby Outdoor Air
Source: Appl Environ Microbiol. 2016 Jun 13;82(13):3822–33. doi: 10.1128/AEM.00610-16 (PMC4907200; doi:10.1128/AEM.00610-16)
Supplement: Supplemental material [file supp_82_13_3822__index.html]

Supplemental material 

# Contribution of Vegetation to the Microbial Composition of Nearby Outdoor Air

## Supplemental material

- Supplemental file 1 -

  Map of the sampling sites where air and plant samples were collected in the San Francisco Bay area (Fig. S1); nonmetric multidimensional scaling plot in two dimensions, constructed from a Bray-Curtis distance matrix of bacterial OTU abundances of all samples on the initial data set, color coded by sample type (Fig. S2); univariate diversity estimators organized by habitat type for bacteria and fungi at a given sampling location (Fig. S3); hierarchical clustering based on the Bray-Curtis dissimilarity index. (Fig. S4); differences based on three dissimilarity indices between habitat type and site (Table S1); estimated sample coverage, observed richness, estimated OTU richness, diversity indices, and ratio of observed to expected richness for upwind air, plant, and downwind air samples (Table S2).

  PDF, 5.2M
