# Supplementary material for: Contribution of Vegetation to the Microbial Composition of Nearby Outdoor Air
Source: Appl Environ Microbiol. 2016 Jun 13;82(13):3822–33. doi: 10.1128/AEM.00610-16 (PMC4907200; doi:10.1128/AEM.00610-16)
Supplement: Supplemental material [file AEM.00610-16_zam999117226so1.pdf]

SUPPLEMENTAL MATERIAL

Contribution of vegetation to the microbial composition of nearby outdoor air

Despoina S. Lymperopoulou,[#] Rachel I. Adams, Steven E. Lindow[#]

Department of Plant & Microbial Biology, University of California, Berkeley, CA 94720, USA

Running Head: Interplay between phyllosphere and outdoor air.

[#]Address correspondence to:

Despoina S. Lymperopoulou, Department of Plant & Microbial Biology, University of California, Berkeley, CA 94720, USA, dlympero@berkeley.edu

Steven E. Lindow, Department of Plant & Microbial Biology, University of California, Berkeley, CA 94720, USA, icelab@berkeley.edu

This file includes:

Supplementary Figures S1 to S4

Supplementary Tables S1 to S2

References

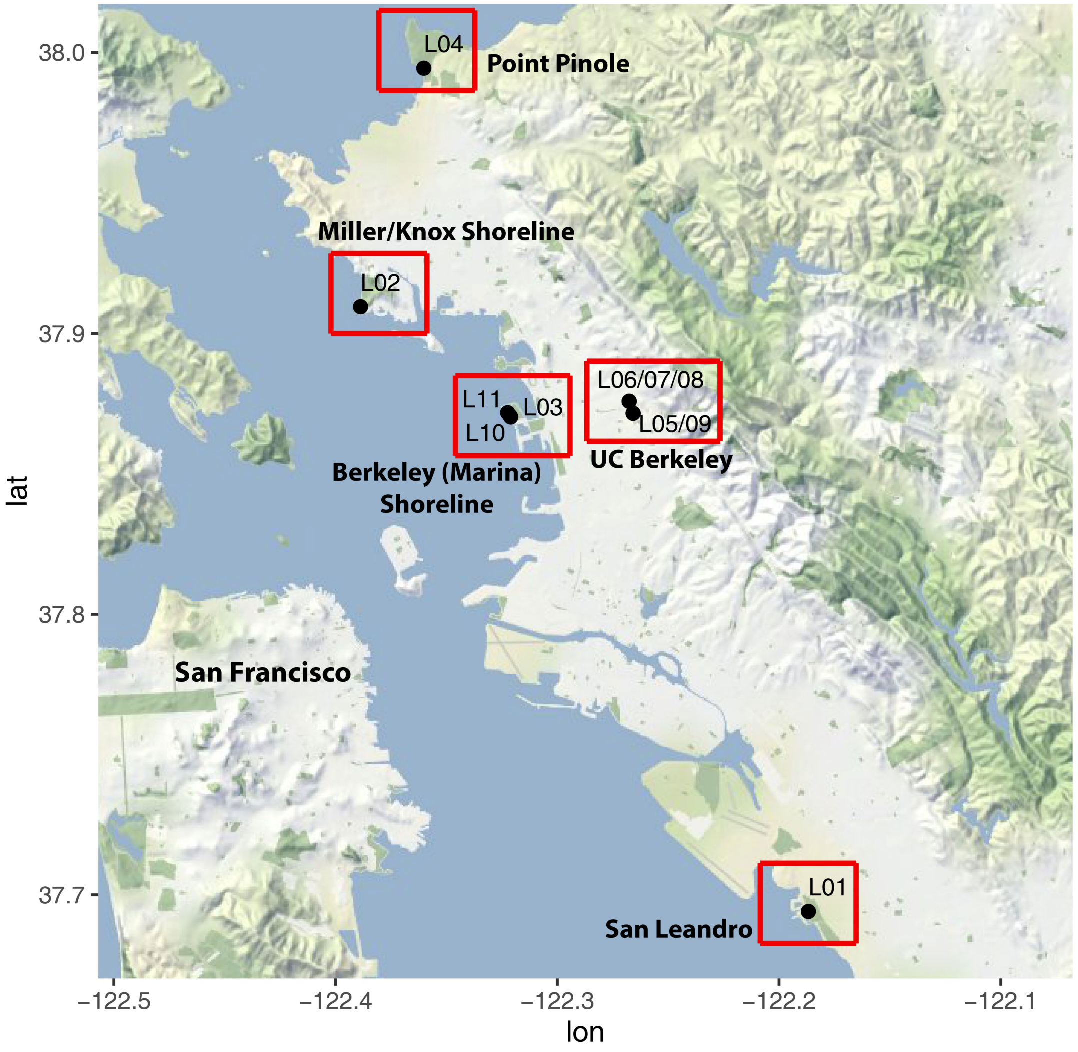

FIG S1 Map of the sampling sites where air and plant samples were collected in the San Francisco Bay area. Coordinates of the locations can be found in Table 1. Upwind and downwind sampling sites are not visualized separately because they cannot be resolved due to their low proximity (< 50 m apart). The map was plotted in R with the package ggmap (1)

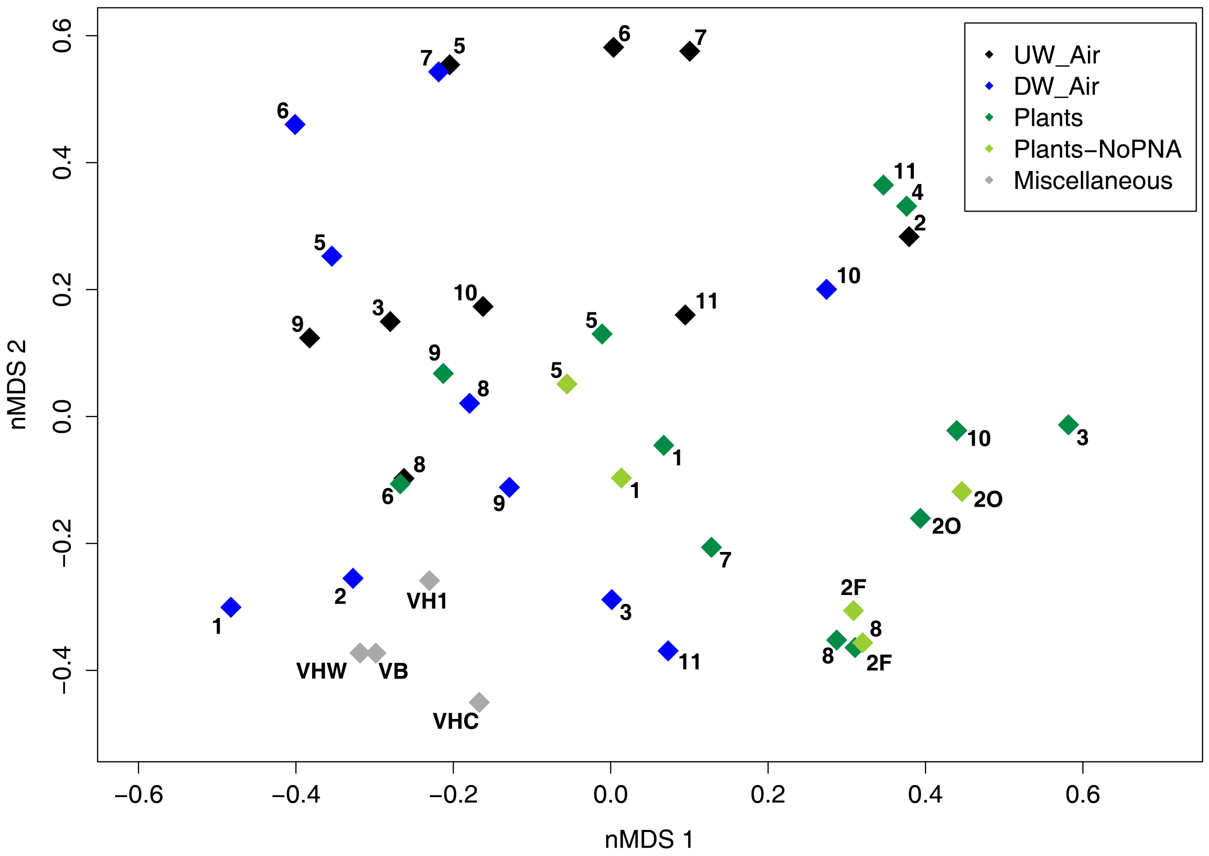

FIG S2 Non-metric multidimensional (nMDS) scaling plot in two dimensions constructed from a Bray-Curtis distance matrix of bacterial OTU abundances of all samples on the initial data set, color-coded by sample type. "Plants - NoPNA" correspond to plant samples that were amplified without the use of PNA and show a nearly identical composition to those amplified with PNA (those labeled "Plants").

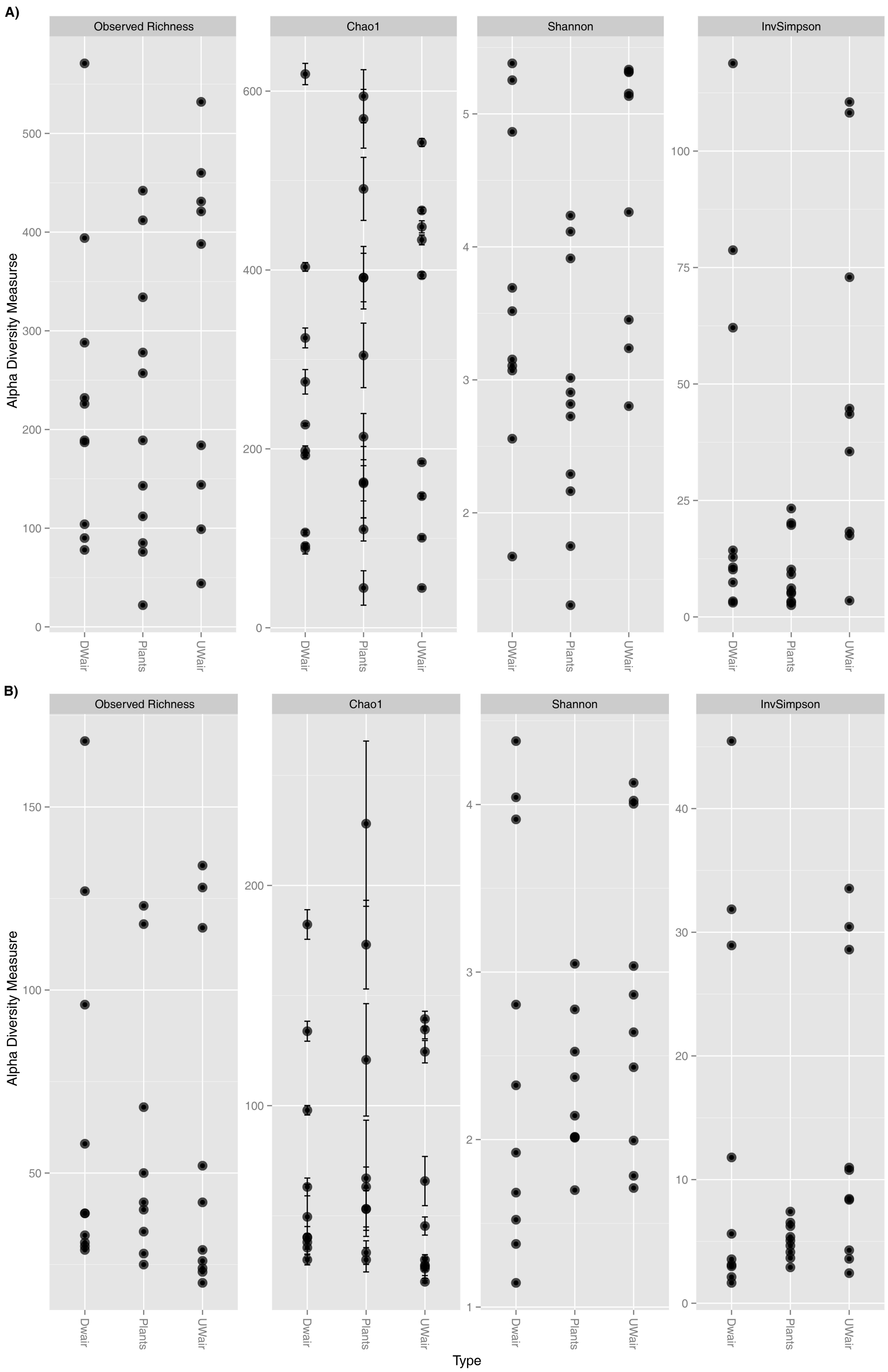

FIG S3 Univariate diversity estimators organized by habitat type (Upwind Air - UWair, Plants, Downwind Air - DWair), for Bacteria (A) and Fungi (B) at a ginen sampling location.

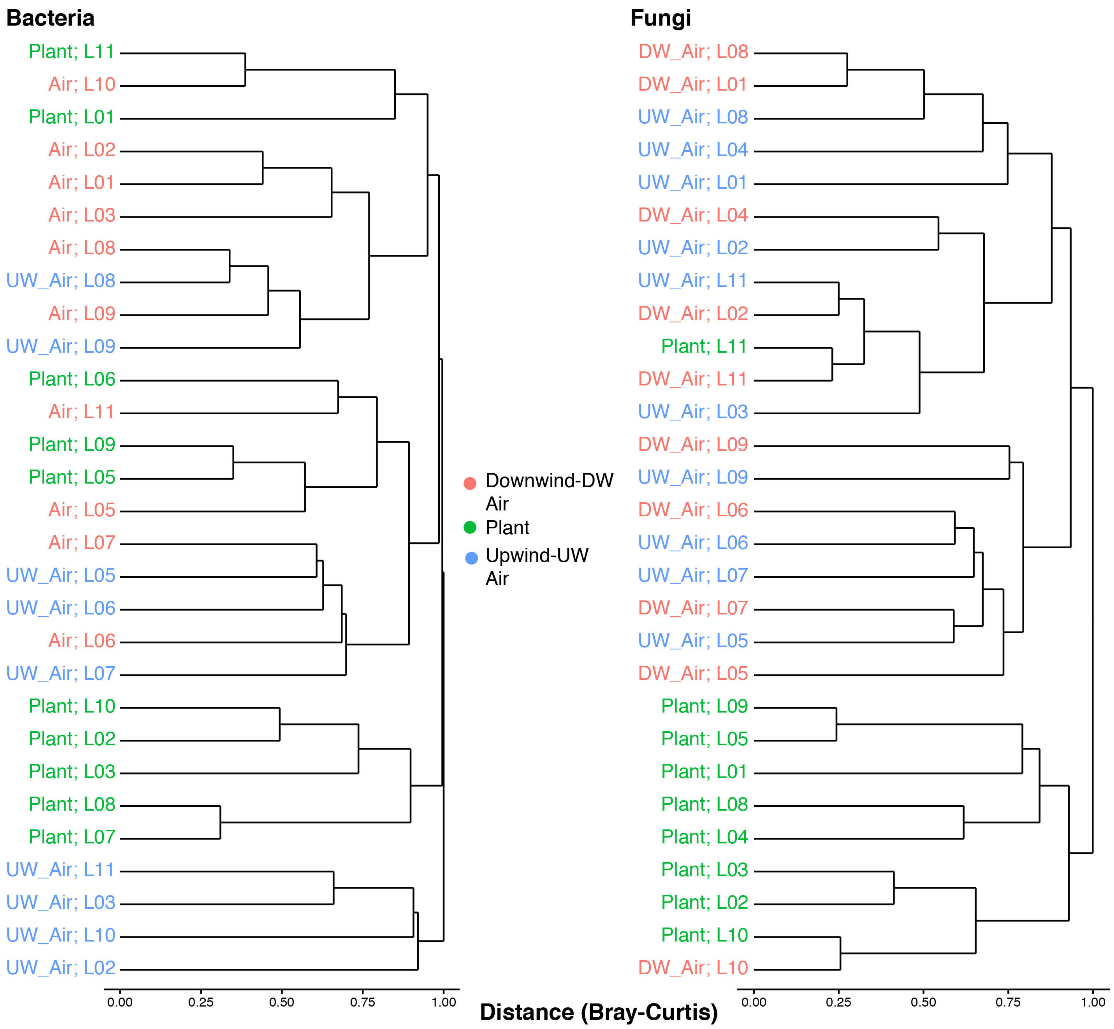

FIG S4 Hierarchical clustering based on the Bray-Curtis dissimilarity index.

TABLE S1 Differences based on three dissimilarity indices, between habitat type (Upwind control, Air, Plants) and Site (location). Significance (p-value) and variation explained (R^2) of the bacterial community composition between the samples determined by analysis of variance based on distance matrices (adonis).

Index		Habitat Type		Site	
		Bacteria	Fungi	Bacteria	Fungi
**Bray-Curtis**					
	p-value	0.001	0.002	0.176	0.969
	R^2	0.14	0.16	0.04	0.02
**Canberra**					
	p-value	0.001	0.001	0.354	0.379
	R^2	0.09	0.09	0.04	0.04
**Jaccard**					
	p-value	0.001	0.002	0.15	0.943
	R^2	0.11	0.12	0.04	0.02

TABLE S2 Estimated sample coverage (Good's C), observed richness (OTUs), estimated OTU richness (S_{Chao1}), diversity indices (Inverse Simpson, Shannon, Berger-Parker), and ratio of observed to expected richness for Upwind Air (VC), Plant (VPP), and Downwind Air (VS) samples. A) Bacteria and B) Fungi.

A) Bacteria

Sample	Good's C	Sobs	Chao1	Inverse Simpson	Shannon (H)	Berger-Parker	Sobs/Chao1	Sobs/Chao1 SD (±)
VC02	99.7%	148	155	3.50	2.81	0.53	95.4%	9.7%
VC03	98.1%	501	531	46.12	5.19	0.12	94.4%	4.6%
VC05	98.4%	446	479	112.48	5.34	0.03	93.2%	5.8%
VC06	99.0%	402	417	114.34	5.33	0.04	96.5%	4.3%
VC07	98.2%	455	493	44.52	5.16	0.13	92.3%	6.1%
VC08	99.9%	100	103	18.42	3.45	0.12	97.6%	9.1%
VC09	99.8%	185	187	35.83	4.26	0.07	99.2%	3.3%
VC10	99.9%	47	52	17.55	3.24	0.15	90.4%	27.6%
VC11	96.1%	636	723	78.14	5.43	0.07	88.0%	6.1%
VPP01	99.1%	95	143	9.22	2.74	0.19	66.2%	25.8%
VPP02	98.3%	158	273	10.28	3.04	0.23	57.9%	21.4%
VPP03	96.7%	286	503	6.26	2.95	0.30	56.9%	15.7%
VPP04	97.5%	215	421	5.37	2.86	0.40	51.1%	18.8%
VPP05	94.0%	500	878	24.44	4.34	0.13	56.9%	11.8%
VPP06	95.4%	382	664	20.73	3.98	0.13	57.5%	13.3%
VPP07	99.0%	79	179	3.32	1.76	0.50	44.1%	27.9%
VPP08	99.7%	24	57	3.09	1.31	0.44	42.1%	41.7%
VPP09	93.4%	532	874	20.71	4.23	0.14	60.9%	10.7%
VPP10	98.7%	122	196	5.09	2.31	0.37	62.2%	22.5%
VPP11	95.9%	326	547	2.59	2.25	0.61	59.6%	13.6%
VS01	99.1%	92	121	3.07	1.70	0.51	76.3%	20.8%
VS02	99.8%	92	95	7.43	3.07	0.33	96.8%	10.3%
VS03	97.7%	309	368	14.48	3.72	0.20	83.9%	9.9%
VS05	95.4%	641	763	65.23	5.32	0.07	84.0%	7.0%
VS06	99.7%	231	235	80.44	4.87	0.05	98.4%	4.1%
VS07	98.9%	408	429	123.16	5.39	0.04	95.2%	5.3%
VS08	99.6%	191	196	12.87	3.52	0.21	97.5%	5.2%
VS09	99.8%	105	109	10.21	3.11	0.21	96.8%	10.3%
VS10	98.1%	243	301	3.39	2.58	0.53	80.7%	12.5%
VS11	99.2%	190	203	10.69	3.16	0.16	93.5%	8.2%

B) Fungi

Sample	Good's C	Sobs	Chao1	Inverse Simpson	Shannon (H)	Berger-Parker	Sobs/Chao1	Sobs/Chao1 SD
VC01	99.3%	46	58	10.93	2.88	0.19	79.3%	48.6%
VC02	99.9%	20	20	8.41	2.43	0.25	100.0%	0.0%
VC03	99.8%	26	28	2.44	1.71	0.63	94.5%	36.1%
VC04	99.7%	31	37	8.52	2.65	0.29	83.8%	52.7%
VC05	97.6%	144	157	35.06	4.16	0.10	91.9%	14.9%
VC06	97.7%	137	152	31.69	4.05	0.10	90.4%	17.2%
VC07	97.8%	128	150	29.82	4.04	0.12	85.4%	24.2%
VC08	99.6%	25	30	4.31	2.00	0.44	83.3%	53.2%
VC09	98.8%	55	78	11.12	3.05	0.20	70.7%	49.7%
VC11	99.8%	23	26	3.60	1.78	0.45	88.5%	51.3%
VP01	98.7%	38	98	3.69	2.03	0.50	38.8%	54.6%
VP02	96.6%	76	158	6.34	2.41	0.30	48.1%	39.7%
VP03	98.3%	50	80	5.46	2.18	0.31	62.5%	47.0%
VP04	97.8%	62	106	7.60	2.58	0.22	58.6%	44.5%
VP05	93.8%	139	247	4.84	2.87	0.44	56.3%	29.3%
VP08	99.4%	26	33	2.91	1.70	0.56	78.8%	53.5%
VP09	92.4%	152	380	6.88	3.17	0.36	40.1%	28.5%
VP10	99.3%	29	36	5.10	2.02	0.33	80.6%	49.0%
VP11	98.5%	45	71	4.18	2.04	0.42	63.8%	49.1%
VS01	99.3%	36	42	1.66	1.16	0.77	85.7%	39.4%
VS02	99.3%	43	47	3.13	1.70	0.48	91.5%	28.3%
VS04	98.7%	62	71	5.68	2.34	0.33	87.0%	28.5%
VS05	94.3%	203	265	50.15	4.49	0.06	76.7%	21.7%
VS06	97.7%	138	157	30.18	4.08	0.11	88.0%	20.5%
VS07	98.8%	104	114	33.14	3.94	0.09	91.6%	21.1%
VS08	99.2%	34	49	2.14	1.39	0.67	69.4%	56.1%
VS09	99.8%	29	30	11.90	2.81	0.20	96.7%	32.9%
VS10	99.3%	41	43	3.58	1.93	0.48	94.6%	22.3%
VS11	98.8%	31	54	2.99	1.53	0.42	57.7%	55.8%

REFERENCES

1. **Kahle D, Wickham H.** 2013. ggmap: A package for spatial visualization with Google Maps and OpenStreetMap. R package version 2.3. See http://CRAN. Rproject. org/package= ggmap.
